# Supplementary material for: Distributionally robust learning-to-rank under the Wasserstein metric
Source: PLoS One. 2023 Mar 30;18(3):e0283574. doi: 10.1371/journal.pone.0283574 (PMC10062629; doi:10.1371/journal.pone.0283574)
Supplement: S1 Appendix — (PDF) [file pone.0283574.s001.pdf]

Distributionally robust learning-to-rank under the Wasserstein metric (Appendix)

Shahabeddin Sotudian[1], Ruidi Chen[1], Ioannis Ch. Paschalidis[1,2*]

1 Department of Electrical and Computer Engineering, Division of Systems Engineering, Boston University, Boston, MA, United States
2 Department of Biomedical Engineering, and Faculty of Computing & Data Sciences, Boston University, Boston, MA, United States

* Corresponding author
Email: yannisp@bu.edu (ICP)

S1 Omitted Proof to Theorem 3.1

For ease of notation, we write $\mathbf{z} \triangleq (\mathbf{x}, \boldsymbol{\theta})$, and the loss function $h(\mathbf{z}) \triangleq \ell(\tilde{\mathbf{B}}\mathbf{z}) = \|\tilde{\mathbf{B}}\mathbf{z}\|_r$, where $\tilde{\mathbf{B}} = (-\boldsymbol{B}', \boldsymbol{I}_K)$ and $\ell(\cdot) = \|\cdot\|_r$. The proof uses a duality theorem for the inner maximization of DRO as an intermediate result, which was originally proposed by [1]. We state it as follows for completeness.

Theorem S1.1 ([1], Theorem 6.3)**.** *Suppose the loss function $h(\mathbf{z})$ is convex in $\mathbf{z} \in \mathcal{Z}$, and the set $\mathcal{Z} \subseteq \mathbb{R}^d$ is closed and convex. Define an ambiguity set Ω around the empirical distribution which is supported on N samples $\mathbf{z}_i, i \in [\![N]\!]$, i.e., $\Omega = \left\{\mathbb{Q} \in \mathcal{P}(\mathcal{Z}) : W_1(\mathbb{Q},\ \hat{\mathbb{P}}_N) \leq \varepsilon\right\}$, where the order-1 Wasserstein metric is induced by some norm $\|\cdot\|$. We have:*

$$\sup_{\mathbb{Q}\in\Omega} \mathbb{E}^{\mathbb{Q}}[h(\mathbf{z})] \leq \kappa\varepsilon + \frac{1}{N}\sum_{i=1}^{N} h(\mathbf{z}_i), \tag{1}$$

where $\kappa = \sup\{\|\boldsymbol{\omega}\|_* :\ h^*(\boldsymbol{\omega}) < \infty\}$, where $\|\cdot\|_*$ stands for the dual norm defined as $\|\boldsymbol{\omega}\|_* \triangleq \sup_{\|\mathbf{z}\|\leq 1} \boldsymbol{\omega}'\mathbf{z}$, and $h^*(\cdot)$ is the convex conjugate function of $h(\mathbf{z})$ defined as: $h^*(\boldsymbol{\omega}) \triangleq \sup_{\mathbf{z}\in\mathcal{Z}} \{\boldsymbol{\omega}'\mathbf{z} - h(\mathbf{z})\}$. Furthermore, (1) becomes an equality when $\mathcal{Z} = \mathbb{R}^d$.

In our loss function, $h(\mathbf{z})$ is convex in $\mathbf{z}$, and thus we can apply Theorem S1.1. The key is to derive the value of κ. Note that,

$$\begin{aligned}
h^*(\boldsymbol{\omega}) &\triangleq \sup_{\mathbf{z}\in\mathbb{R}^{p+K}} \{\boldsymbol{\omega}'\mathbf{z} - h(\mathbf{z})\} = \sup_{\mathbf{z}\in\mathbb{R}^{p+K}} \{\boldsymbol{\omega}'\mathbf{z} - \ell(\tilde{\mathbf{B}}\mathbf{z})\} \\
&= \sup_{\mathbf{z}\in\mathbb{R}^{p+K}} \left\{\boldsymbol{\omega}'\mathbf{z} + \inf_{\boldsymbol{\zeta}\in\mathcal{T}}\{-\boldsymbol{\zeta}'\tilde{\mathbf{B}}\mathbf{z} + \ell^*(\boldsymbol{\zeta})\}\right\} \\
&= \sup_{\mathbf{z}\in\mathbb{R}^{p+K}} \inf_{\boldsymbol{\zeta}\in\mathcal{T}} \left\{\boldsymbol{\omega}'\mathbf{z} - \boldsymbol{\zeta}'\tilde{\mathbf{B}}\mathbf{z} + \ell^*(\boldsymbol{\zeta})\right\} \\
&= \inf_{\boldsymbol{\zeta}\in\mathcal{T}} \sup_{\mathbf{z}\in\mathbb{R}^{p+K}} \left\{(\boldsymbol{\omega}' - \boldsymbol{\zeta}'\tilde{\mathbf{B}})\mathbf{z} + \ell^*(\boldsymbol{\zeta})\right\},
\end{aligned}$$

where $\ell^*(\cdot)$ is the convex conjugate of $\ell(\cdot)$, and $\mathcal{T} \triangleq \{\boldsymbol{\zeta} : \ell^*(\boldsymbol{\zeta}) < \infty\}$. Note that to make $h^*(\boldsymbol{\omega}) < \infty$, it must satisfy $\boldsymbol{\omega}' = \boldsymbol{\zeta}'\tilde{\mathbf{B}}$, otherwise the above inner supremum would achieve ∞. Thus,

$$h^*(\boldsymbol{\omega}) = \inf_{\boldsymbol{\zeta}\in\mathcal{T}} \{\ell^*(\boldsymbol{\zeta})\} < \infty.$$

The regularizer κ can be computed as:

$$\kappa = \sup\{\|\boldsymbol{\omega}\|_s : \ h^*(\boldsymbol{\omega}) < \infty\} = \sup\{\|\tilde{\mathbf{B}}'\boldsymbol{\zeta}\|_s : \ \boldsymbol{\zeta} \in \mathcal{T}\}.$$

The convex conjugate of $\ell(\cdot) = \|\cdot\|_r$ is,

$$\ell^*(\boldsymbol{\zeta}) \triangleq \sup_{\mathbf{t}\in\mathbb{R}^K} \{\boldsymbol{\zeta}'\mathbf{t} - \|\mathbf{t}\|_r\} = \begin{cases} 0, & \text{if } \|\boldsymbol{\zeta}\|_s \leq 1, \\ \infty, & \text{otherwise.} \end{cases}$$

Therefore,

$$\kappa = \sup\{\|\tilde{\mathbf{B}}'\boldsymbol{\zeta}\|_s : \ \boldsymbol{\zeta} \in \mathcal{T}\} = \sup\{\|\tilde{\mathbf{B}}'\boldsymbol{\zeta}\|_s : \ \|\boldsymbol{\zeta}\|_s \leq 1\} = \|\tilde{\mathbf{B}}'\|_s,$$

where the last step follows from the definition of the induced matrix norm. Plugging the value of κ into (1), and replacing the sample average loss by $\frac{1}{\sum_{e=1}^{T} n_e} \sum_{q=1}^{T} \sum_{d=1}^{n_q} \|\boldsymbol{\theta}_d^q - \mathbf{B}'\mathbf{x}_d^q\|_r$, we obtain the desired result.

S2 NDCG Deviation Score Derivation

Let $\mathbb{S}_n = \{(\mathbf{x}_1, y_1), ..., (\mathbf{x}_n, y_n)\}$ be a set of ***sorted*** documents. The NDCG score for $\mathbb{S}_n$ is equal to 1 and can be computed as follows:

$$\frac{1}{\Phi^I(\mathbb{S}_n)} \left(\mu + \frac{y_d}{\log(1 + \pi_d^{-1})} + \frac{y_{\pi_i}}{\log(1+i)} \right) = 1, \quad (2)$$

where π_d^{-1} is the position of document d in $\mathbb{S}_n$, π_i is the index of the document ranked at position i of $\mathbb{S}_n$, Φ^I is the ideal DCG, and μ can be computed as follows:

$$\mu = \sum_{\substack{r=1 \\ r\neq i, \pi_d^{-1}}}^{n} \frac{y_{\pi_r}}{\log(1+r)}.$$

If we switch two documents in $\mathbb{S}_n$, the NDCG will decrease or in some cases may stay the same. For document d, we define the NDCG deviation score vector as $\boldsymbol{\xi}_\Phi = (\lambda_{d1}, \lambda_{d2}, ..., \lambda_{dn_q})$ where λ_{di} is the NDCG score of $\mathbb{S}_n$ when we switch the ***position*** of document d with the ***document*** that is in i-th position of the sorted list $\mathbb{S}_n$. Assume $\hat{\mathbb{S}}_n$ is the document list corresponding to λ_{di}. The NDCG score for $\hat{\mathbb{S}}_n$ can be computed as follows:

$$\lambda_{di} = \frac{1}{\Phi^I(\mathbb{S}_n)} \left(\mu + \frac{y_{\pi_i}}{\log(1 + \pi_d^{-1})} + \frac{y_d}{\log(1+i)} \right). \quad (3)$$

Using (2) and (3), λ_{di} can be formulated as follows:

$$\lambda_{di} = 1 + \frac{\frac{y_d - y_{\pi_i}}{\log(1+i)} + \frac{y_{\pi_i} - y_d}{\log(1+\pi_d^{-1})}}{\Phi^I}.$$

S3 GTD Ablation Study

To gauge the effect of GTD's components on DRMRR performance, we performed the following ablation study. We trained DRMRR on the OHSUMED dataset. We considered two settings: (1) fixed $\boldsymbol{\xi}_\Phi$, and (2) fixed $\boldsymbol{\xi}_D$ and ξ_I. We fixed their values to all-ones vectors. Table S1 summarized performance degradation compared to Table 1 in the paper.

Table S1. GTD Ablation Study.

Setting	NDCG@5	NDCG@10
Fixed $\boldsymbol{\xi}_D$ and ξ_I	-6.65%	-4.59%
Fixed $\boldsymbol{\xi}_\Phi$	-1.33%	-1.56%

S4 DRP data and pre-processing steps

S4.1 Drug Response Prediction data set

Our Drug Response Prediction (DRP) data set [2, 3] contains a total of 332 cell lines (i.e., patients) and 50 drug responses. We used the cell lines which were derived from various cancer tumors such as blood, lung, brain, skin, bone, just to name a few. The data were standardized so that variables lie between zero and one. The goal is to rank drugs based on their response in such way that the most effective drugs are ranked on top of the ranking list. The lists of drugs and cell lines can be found in Tables S2 and S3.

We characterize cell lines with a matrix $\mathbf{C} \in \mathbb{R}^{N_C \times N_G}$, where each row corresponds to one of N_C cell lines. A gene expression vector containing N_G features is used to represent each cell line. Moreover, we represent drug responses to N_D different drugs with another matrix $\mathbf{R} \in [0, 1]^{N_C \times N_D}$. Each row of $\mathbf{R}$ corresponds to a cell line and columns represent the response of that patient to various drugs. Furthermore, drug

Table S2. List of drugs in the DRP data set.

Drugs								
Erlotinib	Sunitinib	PHA-665752	MG-132	Paclitaxel	Cyclopamine	AZ628	Sorafenib	Tozasertib
Imatinib	NVP-TAE684	Crizotinib	Saracatinib	S-Trityl-L-cysteine	Z-LLNle-CHO	Dasatinib	GNF-2	CGP-60474
CGP-082996	A-770041	WH-4-023	WZ-1-84	BI-2536	BMS-536924	BMS-509744	CMK	Pyrimethamine
JW-7-52-1	A-443654	GW843682X	Entinostat	Parthenolide	GSK319347A	TGX221	Bortezomib	XMD8-85
Seliciclib	Salubrinal	Lapatinib	GSK269962A	Doxorubicin	Etoposide	Gemcitabine	Mitomycin-C	Vinorelbine
NSC-87877	Bicalutamide	QS11	CP466722	Midostaurin				

Table S3. List of cell lines in the DRP data set.

Cell lines								
CTV-1	MEC-1	U-698-M	SK-MM-2	NCI-H524	U-87-MG	ES6	IMR-5	NCI-H747
CCRF-CEM	JVM-3	WSU-NHL	EJM	NCI-H82	Becker	EW-3	IST-MES1	NCI-SNU-1
BE-13	HL-60	Ramos-2G6-4C10	L-1236	NCI-H446	D-283MED	EW-12	K5	NCI-SNU-16
EoL-1-cell	MOLM-13	BC-3	L-428	NCI-H2171	GI-1	EW-1	KGN	NCI-SNU-5
GR-ST	PL-21	BC-1	SUP-HD1	NCI-H1694	KALS-1	EW-18	KM12	NEC8
H9	OCI-AML2	SCC-3	L-540	CPC-N	KNS-42	EW-16	KP-N-YN	NH-12
HC-1	OCI-AML5	CTB-1	HDLM-2	NCI-H1876	KS-1	EW-13	KP-N-YS	NOS-1
KINGS-1	MONO-MAC-6	HD-MY-Z	KM-H2	NCI-H2081	NMC-G1	EW-24	KURAMOCHI	OCUB-M
KMOE-2	SIG-M5	SUP-T1	SU-DHL-1	NCI-H1092	ONS-76	RH-1	L-363	OMC-1
J-RT3-T3-5	NOMO-1	OCI-LY-19	DEL	COR-L88	SF126	SK-ES-1	LAN-6	OS-RC-2
LC4-1	OCI-AML3	JJN-3	SUP-M2	COR-L95	AM-38	TC-71	LB1047-RCC	OVCAR-4
ML-2	THP-1	RL	KARPAS-299	HCC-33	CAS-1	A253	LB2241-RCC	PSN1
NKM-1	EM-2	MC116	NCI-H1770	NCI-H1963	KNS-81-FD	ACN	LB771-HNC	RCC10RGB
P30-OHK	NB4	P3HR-1	NCI-H345	NCI-H209	CP66-MEL	ARH-77	LB831-BLC	RKO
TUR	SKM-1	GA-10	NCI-H64	NCI-H2141	LB2518-MEL	BB30-HNC	LB996-RCC	RPMI-6666
RPMI-8866	P31-FUJ	BL-70	LU-139	NCI-H1836	LB373-MEL-D	BB49-HNC	LNCaP-Clone-FGC	RXF393
QIMR-WIL	GDM-1	BL-41	Calu-6	NCI-H69	MZ7-mel	C2BBe1	LS-1034	SCC-15
ATN-1	KG-1	ST486	IST-SL1	NCI-H2227	DJM-1	CGTH-W-1	LS-123	SCH
CESS	KCL-22	HT	IST-SL2	DMS-153	IST-MEL1	COLO-320-HSR	LS-411N	SIMA
A4-Fuk	KU812	SU-DHL-6	LB647-SCLC	DMS-79	MMAC-SF	COLO-684	LS-513	SK-LMS-1
ALL-PO	HEL	DOHH-2	LU-134-A	COLO-668	MZ2-MEL	COLO-824	MFH-ino	SK-N-DZ
KASUMI-1	TF-1	WSU-DLCL2	MS-1	NCI-H526	COLO-829	CW-2	MFM-223	SNU-C1
MOLT-4	OCI-M1	SU-DHL-4	NCI-H510A	NCI-H2196	SK-MEL-2	DSH1	MHH-NB-11	SW684
PF-382	LAMA-84	KARPAS-422	EKVX	NCI-H1385	LOXIMVI	DU-4475	MPP-89	SW872
KE-37	JURL-MK1	NU-DUL-1	HOP-62	SHP-77	UACC-257	EC-GI-10	MRK-nu-1	SW954
ALL-SIL	MEG-01	SU-DHL-8	NCI-H1648	NCI-H211	COLO-800	ECC12	NB10	SW962
DND-41	K-562	CA46	NCI-H1838	D-542MG	HT-144	EHEB	NB12	TE-1
LOUCY	IM-9	EB2	NCI-H1395	SF539	SH-4	ETK-1	NB13	TE-10
RS4-11	SR	NAMALWA	NCI-H2126	SF268	SK-MEL-1	EVSA-T	NB14	TE-12
SUP-B15	Daudi	HH	NCI-H1869	D-247MG	A101D	GCIY	NB17	TE-15
697	DG-75	JVM-2	LXF-289	D-263MG	ES3	GI-ME-N	NB5	TE-5
REH	EB-3	GRANTA-519	LC-1F	D-336MG	ES5	GOTO	NB6	TE-6
BV-173	JiyoyeP-2003	A3-KAW	NCI-H1355	D-392MG	ES7	HCC1187	NB69	TE-8
KOPN-8	MHH-PREB-1	AMO-1	LU-65	D-502MG	EW-11	HCC1599	NB7	TGBC1TKB
ME-1	MN-60	OPM-2	DMS-114	no-10	SJSA-1	HCC2157	NBsusSR	TK10
JM1	no-11	LP-1	NCI-H1581	8-MG-BA	SK-NEP-1	HCC2218	NCI-H226	UACC-812
MEC-1	Raji	MOLP-8	NCI-H23	GB-1	ES8	HCC2998	NCI-H716	VA-ES-BJ
JM1								

responses are in $[0, 1]$ and a lower value indicates lower sensitivity of the cell line to the drug (i.e., a less effective drug). Since we do not have the drug's features such as physical and chemical characteristics; metabolism; and their mechanism of action, we have to use the so-called "one-hot" encoding to represent drugs. Accordingly, we create an indicator variable and the application of the i-th drug will be represented by the i-th unit vector.

By concatenating the one-hot vector representing drug i and the j row vector of $\mathbf{C}$ containing the gene expression for cell line j, we define the $(N_D + N_G)$-dimensional vector $\mathbf{p}_{ij} = (\mathbf{e}_i, \mathbf{c}_j)$, $i = 1, \ldots, N_D$, $j = 1, \ldots, N_C$, to represent a drug-cell line sample. Additionally, we denote the response of drug j in cell line i by r_{ij} which is the (i, j) element of the response matrix $\mathbf{R}$. This process results in $N = N_C \times N_D$ possible combinations of a drug with a cell line. Ultimately, we define the matrix $\mathbf{X} \in \mathbb{R}^{N \times (N_D + N_G)}$ with rows corresponding to drug-cell line pairs. Also, we define $\mathbf{y} = (r_{11}, r_{21}, \ldots, r_{N_D N_C}) \in \mathbb{R}^N$ as the vector of corresponding responses. We use $\{(\mathbf{x}_i, y_i);\ i = 1, \ldots, N\}$ to train the ranking models.

S4.2 Gene selection scheme

DNA microarray technology can simultaneously measure the expression level of many of genes in a biological sample. This can be used to analyze the alterations in a gene expression level in reaction to external stimuli and/or the activation or expression of other genes [4]. In this way, we can examine the level of gene transcription in clinical conditions to monitor the drug response. For further details see [4, 5]. In our drug response data set, genes are represented by approximately 11,000 features. Considering that the number of cell lines is considerably lower than the number of genes (i.e., features), we used a LASSO-based feature selection method [6] to prevent overfitting and select informative genes. In short, this algorithm penalizes the coefficients of the regression variables shrinking some of them to zero. Then, the variables that still have a non-zero coefficient will form the most informative subset of features.

Given the matrix $\mathbf{X}$ and the response vector $\mathbf{y}$, we define a common coefficient vector $\boldsymbol{\nu} = (\boldsymbol{\nu}_1, \boldsymbol{\nu}_2)$ for the rows of $\mathbf{X}$ (i.e., $(\mathbf{e}_i, \mathbf{c}_j)$, $i = 1, \ldots, N_D$, $j = 1, \ldots, N_C$) where $\boldsymbol{\nu}_1 \in \mathbb{R}^{N_D}$ and $\boldsymbol{\nu}_2 \in \mathbb{R}^{N_C}$. Specifically, we considered gene expression vectors and the response values as independent and dependent variables, respectively. The, LASSO

Table S4. List of selected genes.

Gene Encodings								
TUBA1C	KIF21B	PSMB4	COX7A2	FGF2	CLIC1	TSPYL5	DKK1	DHX15
UBE2C	HCLS1	RPN1	NDUFA4	DAB2	LASP1	ID2	EPS8	SRP9
CCNB1	HLA-DRA	ATP6AP1	NDUFA1	SDCBP	IQGAP1	GSTA4	ACSL3	PTGES3
PBK	GNA15	NDUFB11	HEY1	WIPI1	HLA-A	ABCB1	MLPH	CDKN1B
PRR11	CRIP1	GPI	PLP1	SGK1	IFI6	DDC	BDNF	SNRPF
RRM1	SKP1	DBI	TYRP1	BICC1	IFI27	ALDH9A1	MT1X	PFDN2
SRSF2	NHP2	CYP51A1	APOD	PSAP	GBE1	SEPW1	MT2A	HSPH1
ACADM	CCT7	PSMD14	ECM1	WWTR1	SCP2	KDELR2	PLA2G16	UCHL3
RPA3	FBL	MAGED1	QPCT	AVPI1	LXN	ITM2B	RARRES3	NDUFB3
CDKN2C	DDX21	COL5A2	CRYAB	IER3	MGST2	ANKMY2	CYP1B1	MRPL3
NUP37	IMP4	ARNT2	GHR	FHL2	CLIC3	OAT	TXN	HAT1
HN1	DDX47	MYH10	GPR137B	YAP1	PDLIM1	HEBP2	DAD1	PSMD10
EIF4A3	HMGB1	WNT5A	BAMBI	KDELR3	CEACAM6	PRDX4	MARCKS	PSMC3
PIGP	TBCB	TMEM47	AUP1	CAV2	CTSH	TIMP1	SCRN1	PTPLAD1
CD164	YWHAE	MRPS6	MRPL33	PHLDA1	KRT17	GSTO1	CETN2	TAF9
GLRX5	GABARAP	CDC42EP3	HADHB	FOSL1	CSTA	TGFB1I1	KIAA1598	RARS
NEDD8	TMEM97	CD99	ALG8	IGFBP3	SYTL2	HTRA1	MYL9	MRPL13
GLO1	ATP6V0B	PGM1	TMX2	ALDH1A3	ENC1	TMEM158	GGH	TCP1
UBA2	CYB5R3	SH2B3	MRPL49	HMOX1	BMP4	FAM127A	CAP1	ECI2
NARS	PEBP1	TUBB3	TSG101	SOWAHC	AZGP1	PLOD2	GLIPR1	SLC38A2
CAMLG	CYC1	MAP1B	MTHFD2	HEBP1	ASS1	LOX	TMEM14A	WRB
RPS23	BUD31	PLCB4	IARS	LAMB1	TXNIP	TUBB6	ITGAE	IPO7
RPS21	ATP6V1F	SCG5	SLC3A2	TEAD1	DPP4	COL4A2	NDUFA8	PPP1CB
RPL5	YWHAB	SACS	CEBPB	TUFT1	VBP1	AKR1B1	POLE3	HERPUD1
ACTG1	STAU1	GPM6B	SEPHS2	MYO6	ARL6IP1	DFNA5	AP2M1	MAPK6
OAZ1	MAPRE1	IGFBP2	MORF4L1	CYFIP1	GCH1	NCOA4	DPM1	ATP6V1E1
LDHB	PITPNB	EID1	NGRN	PLAT	LTA4H	VAMP7	SNRPB	H3F3B
GMFG	CCT3	NETO2	MANF	PDP1	ERP29	RBBP7	PSMB3	

regression is applied over these variables using the following minimization problem:

$$\min_{\boldsymbol{\nu}} \|\mathbf{y} - \mathbf{X}\boldsymbol{\nu}\|^2 + \mu\|\boldsymbol{\nu}_2\|_1, \tag{4}$$

where $\mu > 0$ is a scalar to control the power of the regularizer. A larger value of μ results in a greater number of coefficients shrunk to zero. We used a recursive feature elimination procedure to drop redundant genes. To that end, we solved (4) using cross-validation to select optimal μ. Five percent of the genes whose corresponding coefficient in $\boldsymbol{\nu}_2$ was among the 5% smaller absolute values were dropped. Problem (4) was reformulated using the remaining features. This procedure was repeated while the validation loss kept decreasing. It is worth mentioning that we examined three values (i.e., 1%, 2.5%, and 5%) for the elimination of the gene features. Nevertheless, the final results were not sensitive to this threshold. Consequently, 5% threshold was used to speed up the process. After conducting the gene selection process, 251 genes were retained. Table S4 presents 251 selected genes after conducting the gene selection process.

S4.3 Drug relevance scores

Our drug responses are in $[0, 1]$. We changed the responses so that a higher response indicates a more effective drug (i.e., one minus the response). In personalized medicine and drug selection, we primarily care about the top few most sensitive drugs. To convert our continuous drug responses to graded ones, we used the following rules: For the k-th cell line, assume P_{80} and P_{90} refer to the 80-th and 90-th percentiles of its drug response values $\{r_{1k}, r_{2k}, \ldots, r_{N_D k}\}$, respectively. Then, the drug relevance score of i-th drug, $\hat{r}_{ik}$ $(i = 1, \ldots, N_D)$, can be calculated as follows:

$$\hat{r}_{ik} = \begin{cases} 2, & \text{if } r_{ik} \geq P_{90} \\ 1, & \text{if } P_{80} \leq r_{ik} < P_{90} \\ 0, & \text{otherwise.} \end{cases}$$

S5 Hyper-parameter optimization

The list of hyper-parameters and their values for all ranking algorithms can be found in Table S5. In this table, η is the learning rate, D_{max} is the maximum depth of a tree, h_{min} is the minimum sum of instance weight (hessian) needed in a leaf, N_T is the

Table S5. The List of Hyper-parameters and Their Values.

Algorithms	Parameters	Values
LambdaMART	h_{min}	1, 5, 10, 50, 100
	η	0.1, 0.01, 0.001
	D_{max}	10, 50, 100
	N_T	10, 100, 1000
$\text{XE-MART}_{\text{NDCG}}$	d_{min}	10, 50, 100
	η	0.1, 0.01, 0.001
	h_{min}	1, 5, 10, 50, 100
	N_T	10, 100, 1000
	ℓ_{max}	10, 100, 200
DRMRR	η	0.1, 0.01, 0.001
	ε	1, 0.1, 0.01, 0.001

number of estimators, d_{min} is the minimal number of data in one leaf, ℓ_{max} is the maximum number of leaves in one tree, and ε is the Wasserstein ball radius. The test sets were exclusively used for evaluating the performance of the algorithms. To find the best result in all experiments, we optimized the algorithm parameters on the validation sets to maximize NDCG@5 and NDCG@10. In DRMRR, we set $\alpha = 50$, $\beta = 0.5$, and $K = 5$. Note that similar to α in $NDCG@\alpha$, K in our model forces the model to focus on the most relevant documents. For simplicity, and since $\alpha = 5$ is the most popular ranking cutoff threshold, we set $K = 5$. Moreover, α enables us to control the maximum score. Therefore, its value does not have a significant effect on the performance. For simplicity, we used the total number of drugs in the DRP data set as the maximum score (i.e., $\alpha = 50$).

We implemented DRMMR with two different loss functions, namely ℓ_1 and ℓ_∞ norms. Since we used convex loss functions, our DRMRR problem was a convex optimization problem. We used mini-batch gradient descent to solve DRMRR. We used the documents in each query as our minibatch. Moreover, we used 50 iterations (i.e., epochs) in all experiments. Based on our validation results, ℓ_∞ was the best loss function for the OHSUMED experiments while ℓ_1 was the best loss function for the DRP experiments. We used a machine with Intel Core i9-9900K 3.6GHz 16MB processor, NVIDIA GeForce RTX 2080 graphics card (8GB), 64 GB memory, and the Linux operating system to run our experiments. We used open-source python packages (i.e., LightGBM [7–9] and XGBoost [10]) to implement the baseline models. The details of hyper-parameter settings and performance of all methods for the five folds can be found in Tables S6, S7, S8, and S9. The values inside the parentheses denote the Standard Deviation (SD) of the corresponding metrics.

S6 Other Competing Methods

In addition to the tree-based baselines, we also compared DRMRR against the state-of-the-art *Transformer-based Neural Ranking (TNR)* model [11] with different loss

Table S6. Hyper-parameter settings and performance of LambdaMART$_{\text{MAP}}$ on OHSUMED and DRP data sets.

Data sets	Folds	NDCG@5	NDCG@10	MAP@5	MAP@10	Parameters			
						h_{min}	η	D_{max}	N_T
OHSUMED	Fold 1	36.22%	37.31%	54.22%	52.97%	10	0.1	10	10
	Fold 2	47.30%	44.95%	67.33%	64.07%	5	0.01	10	10
	Fold 3	43.29%	42.86%	73.16%	66.32%	1	0.01	10	1000
	Fold 4	50.60%	47.87%	74.25%	68.47%	10	0.01	10	10
	Fold 5	48.49%	45.26%	70.75%	68.79%	1	0.01	10	10
	**Average (SD)**	**45.18% (5.07%)**	**43.65% (3.55%)**	**67.94% (7.26%)**	**64.12% (5.83%)**				
DRP	Fold 1	61.06%	66.93%	81.64%	78.69%	1	0.01	10	1000
	Fold 2	58.56%	63.75%	84.53%	78.14%	5	0.001	50	100
	Fold 3	55.97%	60.31%	81.10%	75.70%	5	0.1	50	1000
	Fold 4	58.86%	63.73%	84.26%	77.06%	5	0.01	50	1000
	Fold 5	56.10%	62.24%	84.83%	76.65%	1	0.1	10	100
	**Average (SD)**	**58.11% (1.90%)**	**63.39% (2.17%)**	**83.27% (1.57%)**	**77.25% (1.07%)**				

Table S7. Hyper-parameter settings and performance of LambdaMART$_{\text{NDCG}}$ on OHSUMED and DRP data sets.

Data sets	Folds	NDCG@5	NDCG@10	MAP@5	MAP@10	Parameters			
						h_{min}	η	D_{max}	N_T
OHSUMED	Fold 1	36.80%	35.56%	53.30%	53.54%	5	0.1	10	10
	Fold 2	49.25%	45.85%	72.37%	70.21%	10	0.001	10	100
	Fold 3	41.86%	42.70%	68.35%	63.89%	1	0.1	50	100
	Fold 4	50.38%	48.65%	69.94%	66.80%	10	0.1	10	10
	Fold 5	52.57%	49.27%	78.72%	71.84%	1	0.01	10	1000
	**Average (SD)**	**46.17% (5.91%)**	**44.40% (5.00%)**	**68.53% (8.40%)**	**65.25% (6.47%)**				
DRP	Fold 1	62.25%	66.21%	82.98%	78.43%	5	0.001	10	1000
	Fold 2	60.56%	65.70%	86.35%	78.33%	1	0.01	50	100
	Fold 3	55.16%	58.49%	80.09%	75.56%	1	0.01	10	100
	Fold 4	58.90%	62.23%	82.79%	76.39%	5	0.1	10	1000
	Fold 5	56.78%	61.70%	83.14%	76.06%	1	0.01	10	1000
	**Average (SD)**	**58.73% (2.54%)**	**62.87% (2.83%)**	**83.07% (1.99%)**	**76.95% (1.19%)**				

functions. This model facilitates cross-document interactions through self-attention mechanisms. The model amounts to a permutation-equivariant scoring function since the self-attention operation is permutation-equivariant. We compared DRMRR against three common loss functions, namely Pointwise-RMSE, Ordinal, and RankNet. To train these models, we used the best hyper-parameters suggested in [11]. Accordingly, we set $N = 4$, $H = 2$, $d_h = 512$, $d_p = 0.3$, $L = 240$, and $d_{f_c} = 144$ where N is the number of encoder blocks, H is the number of attention heads, d_h is the hidden dimension, d_p is the dropout probability, L is the list length, and d_{f_c} is the dimension of the linear projection. We trained the networks for 100 epochs with a 0.001 learning rate. Tables S10,S11, and S12 demonstrate the performance of the transformer-based models. As can be seen, the transformer-based neural ranking models achieved acceptable performance. However, since their performances were not comparable to the tree-based baselines (especially on our main application, namely DRP), we did not present their performance in the paper. Please note that NDCG@5 is the most important performance metric in our application.

Table S8. Hyper-parameter settings and performance of XE-MART$_{\mathbf{NDCG}}$ on OHSUMED and DRP data sets.

Data sets	Folds	NDCG@5	NDCG@10	MAP@5	MAP@10	Parameters				
						d_{min}	η	h_{min}	N_T	ℓ_{max}
OHSUMED	Fold 1	37.33%	35.42%	53.10%	51.42%	10	0.01	5	1000	100
	Fold 2	47.19%	47.70%	66.27%	64.02%	10	0.001	5	1000	10
	Fold 3	36.10%	41.20%	59.56%	58.50%	10	0.001	10	100	10
	Fold 4	47.64%	50.00%	72.29%	68.18%	10	0.001	1	100	100
	Fold 5	53.29%	49.62%	75.03%	69.94%	10	0.001	5	10	10
	**Average (SD)**	**44.31% (6.58%)**	**44.79% (5.65%)**	**65.25% (8.08%)**	**62.41% (6.76%)**					
DRP	Fold 1	61.37%	65.97%	82.25%	77.74%	100	0.1	1	1000	10
	Fold 2	61.25%	65.63%	85.87%	79.76%	100	0.01	1	1000	10
	Fold 3	56.10%	60.34%	82.62%	75.72%	100	0.01	1	1000	100
	Fold 4	59.26%	63.88%	84.14%	76.69%	10	0.01	1	1000	10
	Fold 5	58.90%	61.76%	83.63%	77.23%	10	0.001	1	1000	200
	**Average (SD)**	**59.37% (1.92%)**	**63.51% (2.18%)**	**83.70% (1.28%)**	**77.43% (1.34%)**					

Table S9. Hyper-parameter settings and performance of DRMRR on OHSUMED and DRP data sets.

Data sets	Folds	NDCG@5	NDCG@10	MAP@5	MAP@10	Parameters	
						η	ε
OHSUMED	Fold 1	38.62%	37.48%	57.77%	53.68%	0.1	0.1
	Fold 2	50.58%	46.90%	73.79%	66.16%	0.001	1
	Fold 3	49.64%	47.03%	72.90%	67.31%	0.01	1
	Fold 4	42.60%	43.28%	69.68%	62.92%	0.01	1
	Fold 5	57.49%	52.12%	80.06%	76.48%	0.001	1
	**Average (SD)**	**47.79% (6.58%)**	**45.36% (4.84%)**	**70.84% (7.35%)**	**65.31% (7.35%)**		
DRP	Fold 1	68.07%	70.06%	83.90%	79.66%	0.001	0.01
	Fold 2	66.46%	69.21%	86.19%	81.24%	0.001	0.01
	Fold 3	67.10%	70.28%	84.07%	80.38%	0.001	0.001
	Fold 4	68.91%	73.29%	84.48%	81.23%	0.001	0.01
	Fold 5	71.45%	73.52%	86.52%	82.64%	0.001	0.01
	**Average (SD)**	**68.40% (1.74%)**	**71.27% (1.78%)**	**85.03% (1.10%)**	**81.03% (1.00%)**		

Table S10. Hyper-parameter settings and performance of TNR with Ordinal loss function.

Data sets	Folds	NDCG@5	NDCG@10	MAP@5	MAP@10
OHSUMED	Fold 1	34.21%	32.45%	55.12%	54.57%
	Fold 2	52.81%	50.89%	73.86%	68.17%
	Fold 3	48.58%	43.66%	74.75%	70.49%
	Fold 4	43.46%	44.00%	72.98%	69.75%
	Fold 5	44.32%	39.76%	70.13%	66.08%
	**Average (SD)**	44.67% (6.94%)	42.15% (6.74%)	69.37% (8.15%)	65.81% (6.51%)
DRP	Fold 1	58.23%	63.93%	87.07%	81.86%
	Fold 2	55.91%	64.28%	87.39%	79.61%
	Fold 3	55.76%	62.97%	85.64%	78.76%
	Fold 4	54.70%	61.66%	83.91%	78.03%
	Fold 5	52.02%	60.02%	77.08%	72.24%
	**Average (SD)**	55.32% (2.25%)	62.57% (1.75%)	84.22% (4.22%)	78.10% (3.58%)
**Yahoo!**		74.11%	78.53%	89.12%	87.83%

S7 Neural Network (NN) Substitute Models

We used a fully connected network structure with four layers (with ReLU activation functions) as our substitute adversarial model. A uniform variance scaling initializer [12] was utilized as the layer weight initializer. The number of nodes in the layers were 128, 128, 64, and 32, respectively. After each dense layer, we used a dropout layer to randomly drop 20% of input units. The dropout layers help prevent overfitting. The output layer had one node (for XE-MART$_{\mathrm{NDCG}}$ and LambdaMARTs) or K nodes (for DRMRR). Moreover, the *Mean Squared Error (MSE)* between labels and predictions was used in the loss function. We used the Adam [13] optimizer and trained the models by slicing the data into "batches" of size 500, and repeatedly iterating over the entire dataset for 10 epochs. Substitute models were implemented in TensorFlow [14].

S8 Computational Considerations and Limitations

DRMRR solves a convex problem which can be done very efficiently with 1st order gradient methods. Its computational complexity is comparable to the training of leaf nodes in tree models (or the last layer of a neural net model), where a simple regression model is being trained. Further, our loss function is 2-Lipschitz when $r = 2$, suggesting a model complexity comparable to XE$_{\mathrm{NDCG}}$ and superior to LambdaMART (whose model complexity increases with the number of documents per training example). Thus, our model is more efficient (low model complexity) and generalizes better (the generalization error increases with model complexity). However, listwise ranking models can get relatively complex compared to pointwise or pairwise approaches and DRMRR is not an exception.

Table S11. Hyper-parameter settings and performance of TNR with Pointwise-RMSE loss function.

Data sets	Folds	NDCG@5	NDCG@10	MAP@5	MAP@10
OHSUMED	Fold 1	36.23%	34.15%	57.40%	52.21%
	Fold 2	42.22%	42.40%	59.95%	57.66%
	Fold 3	34.66%	32.95%	60.73%	56.78%
	Fold 4	44.74%	44.22%	73.25%	71.35%
	Fold 5	43.38%	42.73%	73.66%	69.23%
	**Average (SD)**	40.25% (4.51%)	39.29% (5.30%)	65.00% (7.82%)	61.44% (8.37%)
DRP	Fold 1	54.82%	61.53%	79.93%	77.19%
	Fold 2	55.63%	63.77%	84.39%	79.50%
	Fold 3	54.45%	61.43%	81.89%	77.16%
	Fold 4	55.02%	61.02%	81.89%	76.86%
	Fold 5	52.37%	58.39%	77.37%	73.27%
	**Average (SD)**	54.46% (1.24%)	61.23% (1.92%)	81.09% (2.61%)	76.80% (2.24%)
Yahoo!		73.69%	78.11%	88.99%	87.72%

Table S12. Hyper-parameter settings and performance of TNR with RankNet loss function.

Data sets	Folds	NDCG@5	NDCG@10	MAP@5	MAP@10
OHSUMED	Fold 1	35.60%	36.27%	62.10%	55.91%
	Fold 2	54.37%	53.38%	73.91%	66.25%
	Fold 3	46.88%	43.81%	74.72%	70.63%
	Fold 4	48.26%	47.90%	73.75%	70.58%
	Fold 5	48.95%	46.23%	80.45%	70.19%
	**Average (SD)**	46.81% (6.88%)	45.52% (6.25%)	72.99% (6.68%)	66.71% (6.31%)
DRP	Fold 1	58.18%	64.23%	86.36%	81.09%
	Fold 2	55.81%	64.18%	85.13%	79.01%
	Fold 3	56.24%	63.25%	85.48%	78.61%
	Fold 4	54.43%	61.72%	84.35%	77.94%
	Fold 5	54.29%	60.47%	78.87%	74.00%
	**Average (SD)**	55.79% (1.58%)	62.77% (1.64%)	84.04% (2.98%)	78.13% (2.59%)
Yahoo!		72.78%	77.42%	88.63%	87.44%

S9 An Example of Algorithm 1

In this section, we present an intuitive toy example to better illustrate the details of Algorithm 1. Fig. S1 demonstrates different steps of our algorithm. Fig. S1-(a), presents the estimated GTD matrix, $\hat{\boldsymbol{\Theta}}^t$, for a test query. In the matrix $\hat{\boldsymbol{\Theta}}^t$, columns correspond to different ranks and rows refer to different documents. Algorithm 1 finds the maximum of i-th column of $\hat{\boldsymbol{\Theta}}^t$ and assigns the corresponding row/document to rank i. Then, it removes the corresponding row/document from $\hat{\boldsymbol{\Theta}}^t$. It repeats this procedure for all ranks.

Step 1

Document	Scores R1	R2	R3	R4	R5	Rank	Document
D1	19.8	23.7	23.1	24.3	21.7	1	
D2	13.9	21.5	19.7	20.5	21.7	2	
D3	14.3	19.6	19.9	23.7	21.9	3	
D4	20.4	23.2	21.6	20.2	24.7	4	
D5	18.8	21.6	20.1	22.0	24.1	5	
D6	22.3	22.4	22.4	20.5	22.5	6	
D7	21.8	23.5	18.6	16.5	20.0	7	
D8	19.3	22.9	20.8	22.3	21.1	8	
D9	17.6	20.3	23.7	24.4	23.4	9	
D10	14.1	19.3	20.4	22.2	22.5	10	

Step 2

Document	Scores R1	R2	R3	R4	R5	Rank	Document
D1	19.8	23.7	23.1	24.3	21.7	1	D6
D2	13.9	21.5	19.7	20.5	21.7	2	
D3	14.3	19.6	19.9	23.7	21.9	3	
D4	20.4	23.2	21.6	20.2	24.7	4	
D5	18.8	21.6	20.1	22.0	24.1	5	
D6	22.3	22.4	22.4	20.5	22.5	6	
D7	21.8	23.5	18.6	16.5	20.0	7	
D8	19.3	22.9	20.8	22.3	21.1	8	
D9	17.6	20.3	23.7	24.4	23.4	9	
D10	14.1	19.3	20.4	22.2	22.5	10	

Step 3

Document	Scores R1	R2	R3	R4	R5	Rank	Document
D1	19.8	23.7	23.1	24.3	21.7	1	D6
D2	13.9	21.5	19.7	20.5	21.7	2	D1
D3	14.3	19.6	19.9	23.7	21.9	3	
D4	20.4	23.2	21.6	20.2	24.7	4	
D5	18.8	21.6	20.1	22.0	24.1	5	
D6	22.3	22.4	22.4	20.5	22.5	6	
D7	21.8	23.5	18.6	16.5	20.0	7	
D8	19.3	22.9	20.8	22.3	21.1	8	
D9	17.6	20.3	23.7	24.4	23.4	9	
D10	14.1	19.3	20.4	22.2	22.5	10	

Step 4

Document	Scores R1	R2	R3	R4	R5	Rank	Document
D1	19.8	23.7	23.1	24.3	21.7	1	D6
D2	13.9	21.5	19.7	20.5	21.7	2	D1
D3	14.3	19.6	19.9	23.7	21.9	3	D9
D4	20.4	23.2	21.6	20.2	24.7	4	
D5	18.8	21.6	20.1	22.0	24.1	5	
D6	22.3	22.4	22.4	20.5	22.5	6	
D7	21.8	23.5	18.6	16.5	20.0	7	
D8	19.3	22.9	20.8	22.3	21.1	8	
D9	17.6	20.3	23.7	24.4	23.4	9	
D10	14.1	19.3	20.4	22.2	22.5	10	

Step 5

Document	Scores R1	R2	R3	R4	R5	Rank	Document
D1	19.8	23.7	23.1	24.3	21.7	1	D6
D2	13.9	21.5	19.7	20.5	21.7	2	D1
D3	14.3	19.6	19.9	23.7	21.9	3	D9
D4	20.4	23.2	21.6	20.2	24.7	4	D3
D5	18.8	21.6	20.1	22.0	24.1	5	
D6	22.3	22.4	22.4	20.5	22.5	6	
D7	21.8	23.5	18.6	16.5	20.0	7	
D8	19.3	22.9	20.8	22.3	21.1	8	
D9	17.6	20.3	23.7	24.4	23.4	9	
D10	14.1	19.3	20.4	22.2	22.5	10	

Step 6

Document	Scores R1	R2	R3	R4	R5	Rank	Document
D1	19.8	23.7	23.1	24.3	21.7	1	D6
D2	13.9	21.5	19.7	20.5	21.7	2	D1
D3	14.3	19.6	19.9	23.7	21.9	3	D9
D4	20.4	23.2	21.6	20.2	24.7	4	D3
D5	18.8	21.6	20.1	22.0	24.1	5	D4
D6	22.3	22.4	22.4	20.5	22.5	6	
D7	21.8	23.5	18.6	16.5	20.0	7	
D8	19.3	22.9	20.8	22.3	21.1	8	
D9	17.6	20.3	23.7	24.4	23.4	9	
D10	14.1	19.3	20.4	22.2	22.5	10	

Step 7

Document	Scores R1	R2	R3	R4	R5	Rank	Document
D1	19.8	23.7	23.1	24.3	21.7	1	D6
D2	13.9	21.5	19.7	20.5	21.7	2	D1
D3	14.3	19.6	19.9	23.7	21.9	3	D9
D4	20.4	23.2	21.6	20.2	24.7	4	D3
D5	18.8	21.6	20.1	22.0	24.1	5	D4
D6	22.3	22.4	22.4	20.5	22.5	6	D7
D7	21.8	23.5	18.6	16.5	20.0	7	:
D8	19.3	22.9	20.8	22.3	21.1	8	
D9	17.6	20.3	23.7	24.4	23.4	9	
D10	14.1	19.3	20.4	22.2	22.5	10	

Fig S1: An Example of DRMRR Score Calculation.

S10 An Example of GTD Vector Calculation

In this section, we present an intuitive toy example to better illustrate the details of GTD vector. Fig. S2,S3, and S4 demonstrates different steps of GTD calculation.

References

1. Esfahani PM, Kuhn D. Data-driven distributionally robust optimization using the Wasserstein metric: Performance guarantees and tractable reformulations. Mathematical Programming. 2018;171(1):115–166.

2. CCLE. Cancer Cell Line Encyclopedia (CCLE); 2021.

3. CTRP. Cancer Therapeutics Response Portal; 2021.

4. Slonim DK, Yanai I. Getting started in gene expression microarray analysis. PLoS Comput Biol. 2009;5(10):e1000543.

Document	Y
D1	1
D2	0
D3	0
D4	2
D5	1
D6	3
D7	4
D8	1
D9	0
D10	0

Document	Perfect Ranking
D7	4
D6	3
D4	2
D1	1
D5	1
D8	1
D2	0
D3	0
D9	0
D10	0

NDCG Deviation Score for D1	
Switch D1 and D7 (Rank #1)	0.79
Switch D1 and D6 (Rank #2)	0.95
Switch D1 and D4 (Rank #3)	0.99
Switch D1 and D1 (Rank #4)	1.00
Switch D1 and D5 (Rank #5)	1.00
Switch D1 and D8 (Rank #6)	1.00
Switch D1 and D2 (Rank #7)	0.99
Switch D1 and D3 (Rank #8)	0.99
Switch D1 and D9 (Rank #9)	0.98
Switch D1 and D10 (Rank #10)	0.98

Fig S2: An Example of GTD Calculation. NDCG deviation score for document 1.

NDCG Deviation Score	Position Deviation Score	Importance Score	GTD - D1
0.79	3.15	0.57	1.41
0.95	5.16	0.57	2.78
0.99	8.05	0.57	4.53
1.00	10.00	0.57	5.68
1.00	5.16	0.57	2.93
1.00	1.91	0.57	1.09
0.99	0.70	0.57	0.40
0.99	0.26	0.57	0.15
0.98	0.10	0.57	0.05
0.98	0.04	0.57	0.02

Fig S3: An Example of GTD Calculation. Position deviation score and importance score for document 1.

Document	Y	GTD Vector									
D1	1	1.41	2.78	4.53	5.68	2.93	1.09	0.40	0.15	0.05	0.02
D2	0	0.23	0.51	0.90	1.52	2.51	3.93	4.89	2.52	0.94	0.34
D3	0	0.14	0.30	0.54	0.92	1.53	2.51	3.94	4.89	2.52	0.94
D4	2	2.86	5.01	6.32	3.23	1.19	0.44	0.16	0.06	0.02	0.01
D5	1	0.84	1.68	2.89	4.57	5.68	2.93	1.08	0.40	0.15	0.05
D6	3	5.28	6.87	3.48	1.25	0.45	0.17	0.06	0.02	0.01	0.00
D7	4	7.34	3.61	1.23	0.41	0.15	0.05	0.02	0.01	0.00	0.00
D8	1	0.50	1.01	1.76	2.93	4.57	5.68	2.92	1.08	0.40	0.15
D9	0	0.08	0.18	0.33	0.56	0.93	1.53	2.52	3.94	4.89	2.52
D10	0	0.05	0.11	0.20	0.34	0.56	0.93	1.54	2.52	3.94	4.89

Fig S4: An Example of GTD Calculation. GTD vectors for all documents.

5. McLachlan GJ, Do KA, Ambroise C. Analyzing microarray gene expression data. John Wiley and Sons. 2005;.

6. Sotudian S, Paschalidis IC. Machine Learning for Pharmacogenomics and Personalized Medicine: A Ranking Model for Drug Sensitivity Prediction. IEEE/ACM Transactions on Computational Biology and Bioinformatics. 2021;.

7. Ke G, Meng Q, Finley T, Wang T, Chen W, Ma W, et al. LightGBM: A highly efficient gradient boosting decision tree. Advances in Neural Information Processing Systems. 2017;30:3146–3154.

8. Meng Q, Ke G, Wang T, Chen W, Ye Q, Ma ZM, et al. A communication-efficient parallel algorithm for decision tree. arXiv preprint arXiv:161101276. 2016;.

9. Zhang H, Si S, Hsieh CJ. GPU-acceleration for Large-scale Tree Boosting. arXiv preprint arXiv:170608359. 2017;.

10. Chen T, Guestrin C. XGBOOST: A scalable tree boosting system. In: Proceedings of the 22nd ACM SIGKDD international conference on knowledge discovery and data mining; 2016. p. 785–794.

11. Pobrotyn P, Bartczak T, Synowiec M, Białobrzeski R, Bojar J. Context-aware learning to rank with self-attention. arXiv preprint arXiv:200510084. 2020;.

12. He K, Zhang X, Ren S, Sun J. Delving deep into rectifiers: Surpassing human-level performance on imagenet classification. In: Proceedings of the IEEE International Conference on Computer Vision; 2015. p. 1026–1034.

13. Kingma DP, Ba J. Adam: A method for stochastic optimization. arXiv preprint arXiv:14126980. 2014;.

14. Chollet F, et al.. Keras; 2015. `https://keras.io`.
